# Correlated single-crystal electronic absorption spectroscopy and X-ray crystallography at NSLS beamline X26-C

**DOI:** 10.1107/S0909049511006315

**Published:** 2011-03-19

**Authors:** Allen M. Orville, Richard Buono, Matt Cowan, Annie Héroux, Grace Shea-McCarthy, Dieter K. Schneider, John M. Skinner, Michael J. Skinner, Deborah Stoner-Ma, Robert M. Sweet

**Affiliations:** a Biology Department, Brookhaven National Laboratory, Upton, NY 11973-5000, USA

**Keywords:** metalloenzymes, cofactors, electronic absorption spectroscopy, Raman spectroscopy

## Abstract

The research philosophy and new capabilities installed at NSLS beamline X26-C to support electronic absorption and Raman spectroscopies coupled with X-ray diffraction are reviewed. This beamline is dedicated full time to multidisciplinary studies with goals that include revealing the relationship between the electronic and atomic structures in macromolecules. The beamline instrumentation has been fully integrated such that optical absorption spectra and X-ray diffraction images are interlaced. Therefore, optical changes induced by X-ray exposure can be correlated with X-ray diffraction data collection. The installation of Raman spectroscopy into the beamline is also briefly reviewed. Data are now routinely generated almost simultaneously from three complementary types of experiments from the same sample. The beamline is available now to the NSLS general user population.

## Abstract 2

The instrumentation and methods available for collecting almost simultaneous single-crystal electronic absorption correlated with X-ray diffraction data at NSLS beamline X26-C are reviewed, as well as a very brief outline of its Raman spectroscopy capability.

## 1. Introduction

Macromolecular crystallography (MX) is the dominant technique used by structural biologists worldwide. Indeed, almost 70000 X-ray crystal structures have been deposited in the Protein Data Bank (PDB) since Kendrew first deposited the structure of sperm-whale myoglobin. It was released in 1976 with the file name 1mbn, 14 years after he received the Nobel Prize in Chemistry with Max Perutz ‘for their studies of the structures of globular proteins’. The impact of the field continues to attract the attention of Nobel Prize committees. They have awarded seven prizes in the past 13 years for work either directly or partially related to MX. Because of this success, the frontier challenges for MX now include structures of trapped reactive intermediates, large macromolecules and viruses, membrane proteins, protein–protein complexes and protein–nucleic acid complexes (NIH, 2008 ▶).

Fundamental insights into these frontier challenges will require an understanding of the complex relationship between electronic and atomic structure. The three-dimensional structure of a molecule is defined by the atomic coordinates for each non-H atom, which are often determined experimentally with X-ray crystallography. In contrast, optical spectroscopy probes the subatomic electronic structure of a molecule, or a subset of atoms within a macromolecule. For example, the electronic absorption spectroscopic analysis of myoglobin provides insights into the iron redox state and its coordination environment. Thus, the electronic structure includes molecular orbitals associated with each atom. Because the overlap or mixing of molecular orbitals results in chemical reactions, macromolecular function is the result of electronic structure. Clearly, the often stated ‘structure–function’ relationship derives from both the atomic and electronic structures of the molecule in question. Consequently, a multi-disciplinary approach focused on probing the electronic, vibrational and atomic structures of macromolecules may provide novel insights of significant relevance to human health.

This is particularly true for macromolecules that utilize cofactors or post-translational modifications for their function. These types of molecules represent a rather large fraction of biology; approximately one-third of all macromolecules expressed by all organisms contain an essential cofactor (Waldron *et al.*, 2009 ▶; Holm *et al.*, 1996 ▶). Those cofactors with color, such as metal ions and/or organic molecules, are present in roughly 11000 entries in the PDB archive (see Table 1 ▶). Usually the spectroscopic signature of these macromolecules changes during catalysis. Furthermore, all biological macromolecules involved in cellular energetics contain cofactors or other modifications. Cofactors and chromophores are also involved or inherent in metal transport and homeostasis, light-sensing systems, the entire fluorescent protein superfamily, nitric oxide synthesis and signaling, redox-state and stress-response systems, and managing the toxicity of reactive oxygen and nitrogen species. Cofactors are also present in RNA and other nucleic-acid-based macromolecules, and often provide essential structural complexity, plasticity and catalytic activity.

Perhaps the best way to correlate electronic and atomic structure is to collect single-crystal spectroscopic and X-ray diffraction data from the same crystal. To that end, some of the first single-crystal microspectrophotometers for use at synchrotron X-ray sources were developed in Europe by Hadfield & Hajdu (1993 ▶) and by Bourgeois *et al.* (2002 ▶). More recent examples include the cryobench and SNBL installations at the ESRF (McGeehan *et al.*, 2009 ▶; Carpentier *et al.*, 2007 ▶; Royant *et al.*, 2007 ▶; Røhr *et al.*, 2010 ▶) and similar efforts at the SLS (Owen *et al.*, 2009 ▶), at the APS (Pearson *et al.*, 2007 ▶; De la Mora-Rey & Wilmot, 2007 ▶) and at the SSRL (Meharenna *et al.*, 2010 ▶). These types of instruments, at the beamline as well as off-line as in the cryobench set-up at the ESRF, have proved to be pivotal in correlating crystal structures to ‘radiation damage’ (Garman, 2010 ▶), or to trap intermediates with characteristic spectroscopic signals for reactive species (reviewed by Pearson & Owen, 2009 ▶; Beitlich *et al.*, 2007 ▶; Bourgeois & Royant, 2005 ▶; De la Mora-Rey & Wilmot, 2007 ▶; Hajdu *et al.*, 2000 ▶). Other reports have documented these methods applied to organic cofactor-containing proteins or those with post-translational modifications: for example, FAD (Major *et al.*, 2009 ▶; Héroux *et al.*, 2009 ▶; Orville *et al.*, 2009 ▶; Churbanova *et al.*, 2010 ▶; Jung *et al.*, 2006 ▶; Barends *et al.*, 2009 ▶; Røhr *et al.*, 2010 ▶), the blue-light sensing FMN-dependent photoreceptor present in the LOV domain (Fedorov *et al.*, 2003 ▶), photoactive yellow protein (Kort *et al.*, 2004 ▶; Rajagopal & Moffat, 2003 ▶; Ren *et al.*, 2001 ▶; Imamoto *et al.*, 2001 ▶), green fluorescent protein (Andresen *et al.*, 2005 ▶; Adam *et al.*, 2008 ▶, 2009 ▶; Rosell & Boxer, 2003 ▶; Lelimousin *et al.*, 2009 ▶) and bacteriorhodopsin (Edman *et al.*, 2004 ▶; Hajdu *et al.*, 2000 ▶; Schertler *et al.*, 1991 ▶). Several metalloproteins have been investigated with correlated studies, including those that contain copper (Wilmot *et al.*, 1999 ▶; Solomon *et al.*, 2004 ▶; Chang *et al.*, 2010 ▶; Pearson *et al.*, 2007 ▶), nickel (Cedervall *et al.*, 2010 ▶), non-heme, mononuclear iron (Adam *et al.*, 2004 ▶; Katona *et al.*, 2007 ▶) or iron–sulfur centers (Karlsson *et al.*, 2000 ▶).

Heme-based enzymes have perhaps the oldest and richest history in protein crystallography (Kendrew *et al.*, 1958 ▶; Perutz, 1963 ▶; Perutz *et al.*, 1968 ▶; Bolton & Perutz, 1970 ▶) and exhibit a molar absorptivity (Table 1 ▶) that is among the highest for all protein cofactors. Research over the past 50 years into the structure and function of heme proteins has produced several iconic results. However, correlated crystallographic and spectroscopic studies are only just now providing critical new insights into the nature of reactive intermediates along the reaction coordinate for only a few of these proteins. For example, high-valent intermediates for several heme-containing proteins have been generated and studied by correlated methods including cytochrome P450_cam_ (Denisov *et al.*, 2005 ▶; Beitlich *et al.*, 2007 ▶; Schlichting *et al.*, 2000 ▶; Makris *et al.*, 2006 ▶), cytochrome *c* peroxidase (Meharenna *et al.*, 2010 ▶), chloroperoxidase (Beitlich *et al.*, 2007 ▶; Kühnel *et al.*, 2007 ▶), horseradish peroxidase (Berglund *et al.*, 2002 ▶), catalase (Jouve *et al.*, 1997 ▶; Gouet *et al.*, 1996 ▶) and the peroxidase activity of myoglobin (Hersleth *et al.*, 2007 ▶). Moreover, the ability to distinguish between Fe(II)–OH_2_, Fe(III)–OH, Fe(III)–O–O(H) and Fe(IV)=O species based on electron density maps is almost impossible in the absence of sub-angstrom resolution data. In contrast, the spectroscopic features of these species are dramatically different. This was recently demonstrated with Fe(III)–NO_2_ and Fe(II)–NO_2_ complexes of myoglobin (Yi *et al.*, 2010 ▶). Consequently, the use of correlated spectroscopic and crystallographic analysis is essential to assign chemical and mechanistic insight(s) to typical resolution structures.

Here we describe our continuing development of beamline X26-C at the National Synchrotron Light Source (NSLS) to support highly correlated studies of single crystals. We have recently integrated several complementary techniques into the beamline so that single-crystal electronic absorption spectroscopy, Raman spectroscopy and X-ray diffraction can be collected from the same sample, and under almost identical experimental conditions. We are about half way through executing our plans to build an offline single-crystal spectroscopy facility adjacent to NSLS beamline X26-C that is likely to include fluorescence spectroscopy with optical absorption and Raman spectroscopies. This will also include polarized optical absorption spectroscopy for detailed anisotropic spectroscopy measurements.

## 2. Beamline X26-C instrumentation for single-crystal spectroscopy

### 2.1. Arrangement of beamline components at X26-C

The NSLS at Brookhaven National Laboratory is a second-generation synchrotron. It started operations with the vacuum ultraviolet (VUV) ring in late 1982 and this was followed by the X-ray ring in 1984. The NSLS has been a very productive facility for almost three decades; but its days are numbered. As we write this review, the NSLS-II is under construction very nearby. The NSLS-II will be a new, state-of-the-art, medium-energy electron storage ring (3 GeV) designed to deliver world-leading intensity and brightness, and will produce X-rays more than 10000 times brighter than the current NSLS (Ozaki *et al.*, 2007 ▶). The NSLS-II is on schedule to begin full operations in 2015. In preparation for this new facility, we are accelerating our development of single-crystal spectroscopy correlated with X-ray diffraction at beamline X26-C. To that end, the research philosophy and focus of this beamline has been redefined recently to support correlated studies on a full-time basis.

The X-ray photons at beamline X26-C emerge from a bending magnet diverting the electron beam in the storage ring. They pass through a channel-cut Si(111) crystal monochromator, and then through a doubly focusing toroidal mirror. The resulting 8–13 keV X-ray beam has an angular divergence of up to 2 mrad with an intensity of approximately 6 × 10^10^ photons s^−1^ in a 200 × 200 µm spot. The final horizontal and vertical beam-defining slits are about 150 mm from the crystal and typically trim the X-ray beam to between 50 and 500 µm in either or both dimensions.

**Figure 1:**
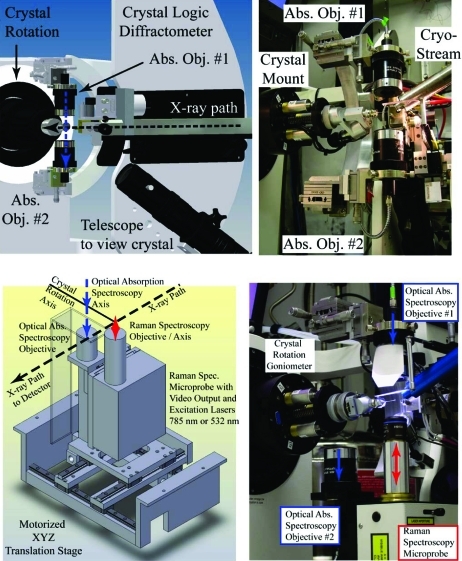
First- and second-phase spectroscopy components installed at beamline X26-C. (Top) A schematic (left) and photograph (right) of the perpendicular alignment of the objectives for electronic absorption spectroscopy correlated with X-ray diffraction. (Bottom) A schematic (left) of the lower objectives for electronic absorption objective #2 and the Raman microprobe head with a close-up view aligned for Raman spectroscopy (right). To achieve phase 2 the lower arm of the arc was replaced with the motorized *XYZ* translation stage.

The right-handed coordinate system inside the hutch at beamline X26-C has the X-ray beam traveling along *x*, the crystal rotation axis parallel to *y*, and the electronic absorption and Raman spectroscopy axis along *z*. Thus, the crystal rotation axis and the X-ray beam define the laboratory horizontal plane (see Fig. 1 ▶). The collimator and final X-ray slits are upstream from the crystal, whereas the ADSC (Poway, CA, USA) Q210R detector occupies the region immediately downstream. The Crystal Logic (Los Angeles, CA, USA) diffractometer carries a telescope to visualize the crystal with a line of sight in the plane of the X-ray beam, from the upstream side, and 35° below the horizontal plane. The diffractometer also carries several attenuators comprised of different thicknesses of aluminium foil and ion gauges to measure the X-ray flux at various points, including just prior to the crystal. Most of the beamline controls are integrated into CBASS (our Python-based beamline control and data collection software) and most events are logged into a database tracking system (Skinner *et al.*, 2006 ▶; Skinner & Sweet, 1998 ▶). The photons for optical absorption spectroscopy travel along the vertical axis and are focused to a point that intersects the crystal rotation point of the diffractometer and the X-ray beam horizontal axis.

### 2.2. Electronic absorption spectroscopy integrated into X-ray diffraction data collection

We adapted the microscopy components from a 4DX-ray Systems AB (Upsala, Sweden) instrument by mounting the objectives to the Crystal Logic diffractometer at beamline X26-C (see Fig. 1 ▶). The *X*, *Y* and *Z* translation slides for the top objective provide a means to align the incident spectroscopy optical axis and its focal point to the crystal rotation axis of the diffractometer and X-ray beam. This is greatly facilitated by visualizing the spectroscopy photon focal spot with an aligned MiTeGen (Ithaca, NY) 400/10 micromesh. In an earlier installation the lower objective was also aligned with manual *XYZ* translation slides. However, in the current installation the lower objective rides on a motorized *XYZ* translation stage that also carries the Raman microprobe (Fig. 1 ▶, bottom). The collection objective is aligned *via* EPICS (Experimental Physics and Industrial Control System; http://www.aps.anl.gov/epics/) and the aligned motor positions are recorded in the beamline control log files and in our experiment tracking database (PXDB) so that one can easily switch between electronic absorption and Raman modes.

The two reflective microscope objectives (telescope objectives) used for optical absorption measurements are based upon the Schwarzschild parabolic mirror design. They were taken from a XSPECTRA system supplied by 4DX-ray Systems AB. They use an all-reflecting principle and are, therefore, free from chromatic aberration in the wavelength range from approximately 150 to 10000 nm. The light is focused to a spot size that depends upon the objective and the diameter of the optical fiber to which it is connected. For example, the incident light photons are focused to approximately a 25 µm-diameter spot using a 50 µm optical fiber, whereas the transmitted photons are collected from approximately a 75 µm-diameter region and focused into a 400 µm optical fiber. The parabolic objectives provide a 24 mm working distance through a 0.4 numerical aperture, which allows for cryocooling and access to the crystal mounting *etc.* The incident light (350–850 nm) is typically from a 75 W Xe research arc lamp (Newport).

We use an Ocean Optics USB 4000 spectrophotometer (Dunedin, FL, USA) containing a 3648-element Toshiba linear CCD detector to collect the optical absorption spectra. The software to control the spectrophotometer in EPICS was initially provided by David Beauregard at the Canadian Light Source, and was locally modified to interface with the X26-C beamline control software (CBASS). The Ocean Optics USB 4000 spectrometer settings, such as integration time (∼50–250 ms), box car (typically 5–10 pixels) and the number of spectra to be averaged, are adjusted to yield maximum sensitivity and signal to noise on the spectrometer CCD.

### 2.3. Experimental steps

**Figure 2:**
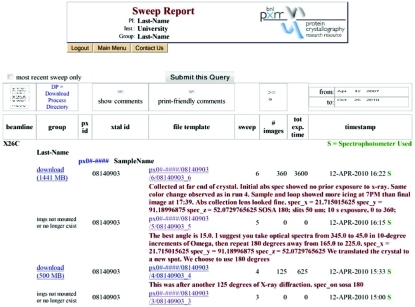
The PXDB was created and is used to track almost all aspects and events during correlated data collection. The front page (not shown) for our PXDB tracking system includes several task-related subheadings. For example, from each of the links one can navigate to metadata related to sample preparation (puck and cane information), beam time request (either in person or Mail-In) and data collection information (Sweep Tools). The Sweep Tools page (illustrated with a screen shot) includes all information logged during data collection from each crystal. This often includes several sweeps for X-ray diffraction screening, finding the optimal crystal orientation for single-crystal spectroscopy, links to the X-ray diffraction images, links to spectroscopic data, and a few summary tools for preliminary spectroscopic analysis that can be of use for decision making while at the beamline.

A typical experiment involves the following steps:

(i) A crystal is mounted and centered manually to the X-ray beam within the cold stream with the Crystal Logic diffractometer.

(ii) 72 digital images of the loop/crystal are recorded, one every 5° around a 360° rotation. At each of the 72 orientations an optical absorption spectrum is also collected according to parameters set for the USB 4000 spectrometer, *e.g.* with an integration time of ∼100 ms per spectrum *etc.*

(iii) The software program CBASS (Skinner *et al.*, 2006 ▶; Skinner & Sweet, 1998 ▶) uses the C3D algorithm (Lavault *et al.*, 2006 ▶) which determines the broadest flat face of the loop/crystal and directs the goniometer to rotate the crystal so that this face is presented to the incident objective lens for spectroscopy. This helps establish the ‘best’ spectroscopy angle for the cryoloop and the crystal. It also has the greatest potential to avoid the cryoloop, which introduces artifacts as it passes through the spectroscopy visible light path. The ‘best’ angle proposed by C3D is recorded in the comments field of the beamline database tracking software, which we call PXDB (see below), and is also passed to CBASS for use during correlated absorption spectroscopy and X-ray diffraction data collection.

(iv) The optical spectra and the crystal images are combined into a side-by-side animated GIF and linked to the PXDB (Figs. 2 ▶ and 3 ▶). Because macromolecular crystals often yield optical spectra that change as a function of rotation angle owing to crystal anisotropy, crystal morphology, refraction, scattering, *etc.*, an interactive Java movie application is also prepared that provides a means to examine the spectra and the crystal image as a function of rotation angle. In the animated GIF and the interactive Java movie the absorption spectrum is correlated to the crystal image from the perspective of the spectroscopy photons passing through the crystal (*e.g.* offset by 90° + 35° = 125° owing to the offset of the crystal viewing telescope 35° below the X-ray plane). Based upon inspection of the Java movie, the user may select an alternative angle for correlated spectroscopic and X-ray data collection.

**Figure 4:**
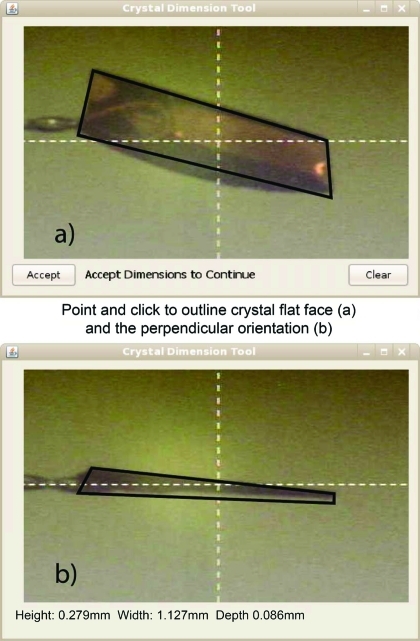
Snapshots of the JAVA application built to quickly estimate the crystal dimensions. The ‘Best’ orientation (top) is used to measure the broadest features of the crystal (solid black lines) by using a mouse to point and click at four points in the image. After one accepts these measurements, the crystal is rotated by 90° in order to obtain a good estimate of the crystal thickness. The combination of these measurement yields the best estimate for all three dimensions of the crystal.

**Figure 5:**
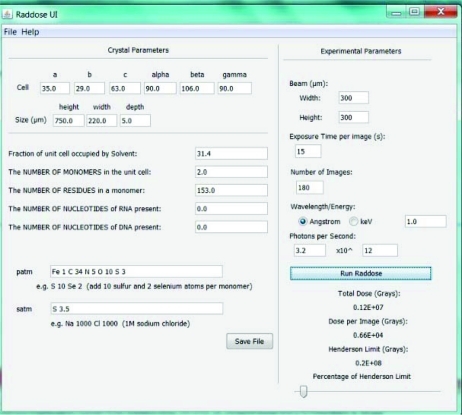
A simple user interface for *RADDOSE* (Paithankar *et al.*, 2009 ▶) was created. This is used to help estimate and/or quantify the extent of X-ray dose (absorbed energy per unit mass) at beamline X26-C. The program *RADDOSE* calculates the dose by using the macromolecular crystal absorption cross section, the X-ray energy, beam parameters, chemical composition and the physical dimensions of the crystal.

**Figure 6:**
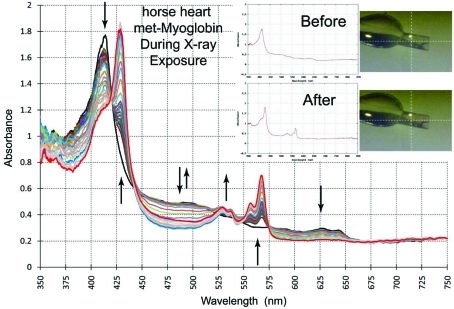
An example of X-ray-induced spectroscopic changes in a single crystal of horse heart met-myoglobin similar to that reported by Yi *et al.* (2010 ▶). The data collection strategy was as outlined in the text. The inset shows the ‘Best’ orientation for the crystal before and after the X-ray diffraction data were collected. The spectra illustrated in the nested set of curves were measured after every 20 s exposure for 15 images and then after every tenth image through 180 total images.

(v) An option to measure the crystal shape and dimensions is available from the Java interactive movie (see Fig. 4 ▶). This takes advantage of the C3D calculation of the flat face for the loop and/or crystal. For example, the scientist uses a mouse to click four corners of the crystal as viewed in its ‘best’ or flattest face orientation. Then, after accepting the trace of the crystal, the view at 90° to the first orientation is presented and the scientist is prompted to trace the crystal again. This yields the three crystal dimensions, which are written into the PXDB log file and used for *RADDOSE* (see below).

(vi) After measuring the crystal dimensions the scientist has the option to execute our *RADDOSE* (Paithankar *et al.*, 2009 ▶) graphical user interface (GUI) as a means of entering additional information for use by the program to estimate the X-ray dose rate (Fig. 5 ▶). Our Java-based GUI emulates the GUI at beamline X10SA at the Swiss Light Source (http://x10sa.web.psi.ch/d_coll/raddose_tk_help.html). The *RADDOSE* information gathered by the GUI is stored in PXDB and used in concert with observed experimental parameters to automatically record radiation dose in the spectra file headers during correlated X-ray diffraction and spectroscopy runs. As part of the beamline preparation prior to data collection the incident X-ray intensity is measured with a calibrated pin-diode placed at the sample location. These measurements are carried out at several X-ray energies, with a variety of beam dimensions, and correlated to the ring current and the ionization gauges located upstream and very close to the sample.

(vii) We collect correlated electronic absorption spectroscopy between almost every X-ray diffraction image at beamline X26-C (Fig. 6 ▶). The crystal is first rotated to either the ‘best’ or the user-selected angle, a spectrum is recorded and plotted in a terminal window. The crystal then rotates to the desired goniometer angle to start the X-ray diffraction data collection. During the readout loop of the X-ray detector and while the X-ray shutter is closed, (*a*) the crystal is rotated back to the ‘best’ or selected orientation, (*b*) another optical spectrum is recorded, and (*c*) the result is plotted as an overlay on the first spectrum. The CBASS program then continues executing the X-ray diffraction data collection strategy at the appropriate rotation angle. This method automatically measures optical spectra after every image for the first 15 images (a program variable that is easily changed), and then after every subsequent tenth image (another program variable). Thus, a family of optical spectra is obtained as a function of X-ray exposure, wherein each spectrum differs from the others by the cumulative X-ray exposure established by the diffraction data collection strategy. At the end of the X-ray diffraction data collection the resulting optical data are linked to the PXDB in several file formats. This includes a means to extract changes in the spectra as a function of wavelength and X-ray exposure time (or dose). Typically the volume of the crystal exposed to the X-ray beam at X26-C is several-fold larger than the volume probed by the 25 µm-diameter spectroscopy beam. Therefore, the spectroscopy photons only probe a region of the crystal that has been exposed to X-rays. However, some crystals may be larger than the X-ray beam in all dimensions. Thus, it is possible that the spectroscopy photons will travel through unexposed as well as exposed regions of the crystal. Under these latter circumstances the absolute correlation of spectroscopic changes as a function of X-ray exposure is less well defined.

(viii) Within the ‘Spectroscopy’ tab of CBASS there is also an option to collect relatively rapid full or single-wavelength spectra from a stationary crystal. The routine is linked to the final X-ray shutter and thus provides an opportunity to measure kinetics, for example, as a function of X-ray dose. Results of this nature have been reported recently by Orville *et al.* (2009 ▶). This feature is also useful for situations where short-lived radical species may be generated by X-ray exposure.

(ix) The optical data are collated and processed for submission to the PDB with the atomic coordinates and structure factors related to the structure determined from the particular crystal. Therefore, the family of absorption spectra that were obtained during the X-ray diffraction data collection is ultimately linked to the atomic coordinates submitted upon publication of the results. We are striving to include as much relevant information as possible about the experiment in the header of the spectroscopy files for deposition in the PDB. The header includes crystal rotation angle, total X-ray exposure time and dose, and spectrometer set-up parameters such as box car, averaging and integration time. Data of this type are most useful for redox-active systems since they are most likely to react with solvated electrons during the X-ray diffraction data collection. As indicated in Table 1 ▶, this could be a significant fraction of structures currently deposited in the PDB archive.

### 2.4. Raman spectroscopy at beamline X26-C

**Figure 7:**
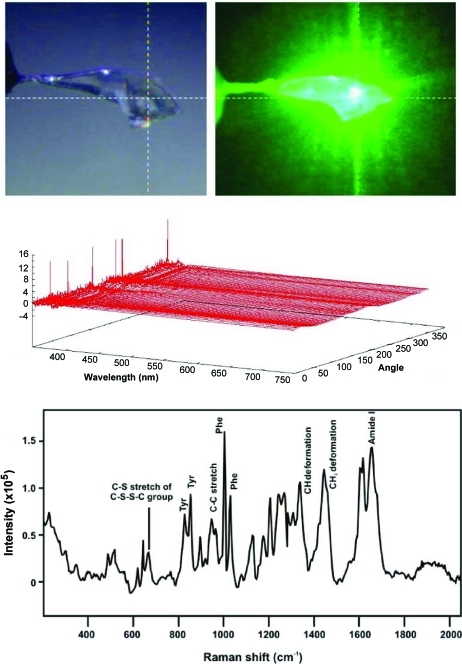
An example of single-crystal electronic absorption and Raman spectroscopy collected from the same Zn^2+^ insulin crystal (Stoner-Ma *et al.*, 2011 ▶). The top two panels show the crystal illuminated with visible light (left) and with the 532 nm laser (right). The middle panel shows the 72 electronic absorption spectra as a function of crystal rotation angle. The bottom panel shows the Raman spectra collected using 6 mW of 532 nm laser excitation and 50 s total acquisition time. This particular crystal morphology has less dependence on finding the ‘best’ or flattest face.

We have also recently installed single-crystal Raman spectroscopy at beamline X26-C. A very brief summary follows here; however, a more comprehensive report has just been published (Stoner-Ma *et al.*, 2011 ▶). A small portion of the Raman instrumentation is illustrated in the bottom portion of Fig. 1 ▶. The system, from Horiba Jobin-Yvon Inc., consists of (*a*) two diode lasers (532 nm and 785 nm), (*b*) two Raman probe heads, each containing an edge filter specific for one of the laser excitation energies, (*c*) an IHR 550 spectrometer, and (*d*) a Synapse CCD detector. We collect data in backscatter geometry and use optical fibers to connect the Raman lasers to the probe heads and back to the spectrometer. The Raman instrumentation is currently controlled by the Windows-based *LabSpec* software provided by Horiba-JY. However, in the near term the Raman spectroscopy experiment will be controlled over a TCP socket interface with CBASS. The intent is to better integrate Raman spectroscopy, electronic absorption spectroscopy and X-ray diffraction data collection strategies. In the meantime we now routinely collect vibrational spectra before and after X-ray exposure. For example, Fig. 7 ▶ shows optical absorption and Raman spectra from Zn^2+^ insulin crystals at 100 K. The Raman features observed are very similar to those reported earlier (Yu *et al.*, 1972 ▶). In the majority of cases examined to date, the baseline of the Raman spectrum is altered slightly by exposure to X-rays. Fortunately, the overall shape of the baseline typically does not change and the sample’s signal-to-noise level is similar for spectra taken before and after X-ray exposure. Occasionally, much larger changes to the Raman spectrum are observed after X-ray exposure. However, these cases appear to be correlated to crystals that also experience significant changes in optical spectra during X-ray exposure. Thus, they may also fluoresce more and/or experience changes in resonance enhancement of the Raman spectrum. Nevertheless, the application of three complementary methods to the same sample is clearly valuable to these types of samples.

### 2.5. Database integration and data tracking at beamline X26-C

We communicate frequently with our collaborators and clients, as well as keep records of all the work carried out at beamline X26-C. To meet these needs and to simplify work for all of our users and staff, a project-management system is used that is integrated with CBASS and is accessible through the web. This system is called PXDB (Fig. 2 ▶). It is based on the relational database management system, Postgres. The PXDB’s web-based interface relies heavily on Javascript, Perl and Python for dynamic content. CBASS interfaces to PXDB through a Python/Postgres library. For example, as CBASS controls the instruments for data collection, it writes HTML files that are made accessible from PXDB sweep queries. Almost all major events and/or parameters of the synchrotron, beamline and diffractometer are recorded. Consequently, the PXDB is fully integrated into most aspects of correlated spectroscopy and X-ray diffraction data collection at beamline X26-C. CBASS creates directories of the form ./[PXID]/[XtalID]/sweep_number/ automatically, into which it stores the resulting X-ray diffraction images and spectroscopy information. The ‘Sweep number’ is determined and iterated as necessary, based upon the record of previous data sweeps performed on the sample as stored in the PXDB. Because it is web-based, the PXDB also serves as a portal for remote scientists to monitor and obtain their data in near real time. This includes X-ray diffraction data and the JAVA animations for displaying and analyzing the single-crystal electronic absorption spectra.

## 3. Summary

Beamline X26-C is dedicated to correlated spectroscopic and X-ray diffraction studies. It is available full time to the general user community and supports electronic absorption and Raman spectroscopies coupled with X-ray diffraction. The complete integration of single-crystal electronic absorption spectroscopy into X-ray diffraction data collection provides new opportunities to the macromolecular crystallography field. For example, spectroscopic changes that may occur as a function of X-ray exposure can serve as a beacon from which new strategies for data collection can be formulated. To that end, several crystals are likely to be used, and the resulting data merged on the basis of the total X-ray dose and/or the observed spectroscopic perturbation. In this way the resulting structures are more accurately ascribed to, for example, the oxidized or reduced state for a given metal center. Furthermore, the deposition to and release of single-crystal spectroscopic data with the atomic coordinates by the PDB further supports and enriches the archive.

## Figures and Tables

**Figure 3:**
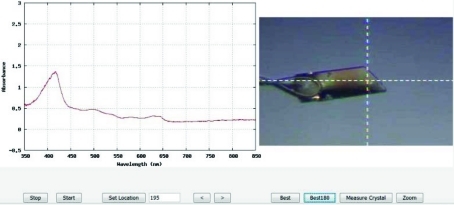
A snapshot of the JAVA interactive window for real-time analysis of single-crystal electronic absorption spectroscopy at beamline X26-C. The buttons along the bottom control the playback of the animation as well as display the C3D analysis of the ‘Best’ and ‘Best180’ images and spectra from the crystal. In this particular case the Best180 image and spectra yield a goniometer setting of 195°. The Measure Crystal tab on the lower right provides the ability to quickly estimate the crystal dimensions.

**Table 1:** Summary of selected cofactors within structures deposited in the PDB archive (June 2010)

Prototypical protein example	Cofactor or chromophore	λ_max_ (nm)	Typical ∊ (m*M*^−1^ cm^−1^)	PDB ligand ID or name	No. of PDB structures
Aspartate aminotransferase	Pyridoxal-5′-phosphate	350, 412	6.6, 9.5	PLP	568
Lactate dehydrogenase	NAD(H) or NADP(H)	340	6.2	NAD, NDP, NAP	1480
Dihydrofolate reductase	Pterin	350	4.5	Pterin	242
*p*-Hydroxybenzoate hydroxylase	FAD or FMN	450	11	FAD, FMN, flavin	1466
Myoglobin	Heme	409	170	Hem	2262
Mn superoxide dismutase	Mn(III)-O/N	480	0.91	Mn	1395
Rubredoxin	Fe (non-heme)	430–500	3–5	Fe	766
Ferredoxin	Fe_2_/S_2_ cluster	∼400	30.6	FeS	304
High potential iron protein	Fe_4_/S_4_ cluster	∼400	15	SF4	388
Methylmalonyl-CoA mutase	Co or B_12_	360	27.5	Cobalt	355
Urease	Ni(II)-O/N	407	0.21	Nickel	461
Azurin or plastocyanin	Cu	600	5	Cu	752
Photosynthetic reaction center	Chlorophyll	465, 665	>100	BCL, BPH, *etc.*	147
Bacteriorhodopsin	Retinal	480	27	Ret	111
Green fluorescent protein	Post-translation modification	350–700	6–94.5	Luminescent proteins	218
